# Telomere‐to‐telomere CHM13 reference reveals missing truth variants and improves deep learning‐based variant calling in long‐read sequencing data

**DOI:** 10.1002/qub2.70051

**Published:** 2026-08-18

**Authors:** Zhenxian Zheng, Minggao He, Xian Yu, Lei Chen, Junzhe Li, Jingcheng Zhang, Ruibang Luo

**Affiliations:** ^1^ School of Computing and Data Science The University of Hong Kong Hong Kong China

**Keywords:** long‐read sequencing, T2T‐CHM13, variant calling

## Abstract

Although long‐read sequencing enables comprehensive genomic analysis, its potential is hindered by its reliance on the incomplete GRCh38 reference genome. GRCh38’s unresolved bases, structural errors, and haplotype mosaics introduce reference biases, leading to missed variants and false‐positive calls. This limitation creates a circular dependency because benchmarks derived from GRCh38 exclude about 15% of complex genomic regions, thereby restricting the training and evaluation of state‐of‐the‐art variant callers. The complete telomere‐to‐telomere (T2T‐CHM13) assembly resolves these gaps and provides an accurate genome‐wide coordinate system. To bridge this transition, we present a variant training framework that integrates high‐confidence variants from both GRCh38 and T2T‐CHM13. Our hybrid training strategy combines rigorous variant filtering and multi‐caller aggregation to produce more reliable variants for model training. This mixed‐reference model demonstrates superior accuracy across sequencing technologies, coverage depths, and diverse samples. Notably, the model trained solely on a single CHM13‐specific sample outperformed models trained on multiple GRCh38‐specific samples on the Oxford Nanopore Technologies platform. Our findings not only confirm the advantages of complete reference genomes but also reveal new challenges and opportunities for variant discovery in complex genomic regions.

## 1 INTRODUCTION

The advent of long‐read sequencing has theoretically unlocked comprehensive variant detection across the genome [1], encompassing accurate small variant [2, 3] and structural variation detection [4, 5, 6, 7]. However, the full potential of this technology is currently limited by its reliance on the GRCh38 reference assembly [8]. Despite serving as the community standard, GRCh38 is fundamentally incomplete; it contains roughly 200 million bases of gaps, misassembled segmental duplications, and creates an artificial mosaic of haplotypes [9]. These limitations introduce significant reference bias during read mapping, particularly in complex genomic regions, resulting in missed variants and tens of thousands of spurious calls per sample [10].

This reliance on an incomplete reference genome creates a circular dependency that propagates beyond read alignment to affect the foundational benchmarks used for training and validating variant callers. State‐of‐the‐art deep learning‐based methods—such as the DeepVariant frameworks [2, 11, 12] and Clair3 frameworks [3, 13, 14, 15, 16, 17]—are predominantly trained on Genome in a Bottle (GIAB) benchmark sets derived from GRCh38. Although GIAB has achieved high coverage of the GRCh38 autosomal genome (about 94%), its benchmarks remain constrained to regions where short‐read technologies yield concordant calls. As a result, substantial portions of the genome—including medically relevant genes, pericentromeric regions, and satellite repeats—are systematically excluded from model training and evaluation. For example, recent comparisons with the T2T‐HG002 assembly indicate that 15.3% of the genome [18], enriched in complex variation, is absent from the standard GIAB v4.2.1 benchmark [19]. When models are trained solely on these comparatively less challenging genomic regions, their accuracy in complex nonreference regions remains unverified, perpetuating a blind spot in genomic variant analysis [20, 21].

Addressing these shortcomings, the complete telomere‐to‐telomere (T2T‐CHM13) assembly represents a transformative milestone [10, 22, 23]. By uncovering more than 100 million previously missing base pairs and rectifying thousands of structural errors, T2T‐CHM13 establishes the first truly gapless genomic coordinate system. Furthermore, the concurrent development of the T2T‐HG002 diploid assembly has catalyzed a paradigm shift from variant‐centric to genome‐centric benchmarking, enabling, for the first time, an unbiased evaluation of variant calling performance across the entire genome.

In this study, we introduced a variant training framework designed to overcome the limitations of single‐reference genome training by leveraging high‐confidence variants derived from both the GRCh38 and T2T‐CHM13 references. Our approach integrates a rigorously filtered set of variants from each assembly, incorporating steps to exclude low‐confidence calls, resolve inconsistent representations, and aggregate results from multiple callers to enhance training reliability. When applied to Clair3 models trained on GRCh38‐specific, T2T‐CHM13‐specific, and mixed‐reference variant sets, the combined model demonstrated improved robustness and accuracy across sequencing technologies, coverage depths, and diverse sample sets. Notably, the mixed model outperformed the GRCh38‐specific baseline even when evaluated on the GRCh38 reference itself, indicating its ability to learn a more generalized representation of genetic variation. Furthermore, our analysis highlights that although T2T‐CHM13 offers a more complete genomic landscape, it also introduces variant‐calling challenges in newly accessible complex regions, underscoring the need for continued refinement in these areas.

## 2 RESULTS

### 2.1 Overview of the cross‐reference model training and benchmarking

To comprehensively evaluate our method, we implemented a rigorous model training strategy utilizing both the GRCh38 and T2T‐CHM13 reference assemblies. We developed three pretrained models for comparison: (1) a GRCh38‐specific baseline (the model trained on GRCh38 reference genome), trained on multiple GIAB datasets; (2) a CHM13‐specific model (the model trained on T2T‐CHM13 reference, using the GIAB HG002 sample), designed to assess the performance gains attributable to the gapless assembly; and (3) a model trained on both T2T‐CHM13 and GRCh38 data (the mixed‐truth model), developed to investigate the synergistic effects of combining training data from both references.

For performance evaluation, we benchmarked our models using the chromosome 20 data from HG001–HG004. To ensure unbiased evaluation, HG004 and HG003 served as a strictly held‐out sample, and chromosome 20 was explicitly excluded from all training sets to test generalization. Benchmarking was performed on both Oxford Nanopore Technologies (ONT) and Pacific Biosciences (PacBio) platforms using identical settings. We begin by comparing the variant distributions between the GRCh38 and T2T‐CHM13 truth sets, followed by a performance evaluation of the models across different sequencing platforms and coverages.

### 2.2 Analysis of the unified variants between two different genomes

We analyzed the unified variants between the T2T‐CHM13 and GRCh38 genomes to assess the complexity of the truth sets. Variants with little to no read support, particularly those lacking alternate allele reads in alignments, are challenging for variant callers to detect because these tools typically rely on aligned reads within a genomic context to make confident calls. The proportion of such low‐read‐support variants thus serves as an indirect indicator of genome complexity and reference quality. To better evaluate the evidence for each reference genome, we applied Repun [24]—a haplotype‐aware variant representation unification method—to minimize the effects of inconsistent variant representations between the truth variant call format (VCF) files and input alignments.

As shown in Supporting Information S1: Table S1, Repun identified unified variants using a minimum variant allele fraction threshold of 5%. Using 50× ONT HG002 whole‐genome sequencing (WGS) data, we classified 993 single‐nucleotide polymorphisms (SNPs) and 1051 indels as low‐confidence variants in the GRCh38 reference genome. In contrast, the T2T‐CHM13 assembly exhibited a markedly higher number of low‐confidence variants, with 11,258 SNPs and 21,748 indels. A similar trend was observed with PacBio sequencing data [25]: for HG002 50× WGS, 8779 SNPs and 7892 indels were missing in T2T‐CHM13, compared to 1687 SNPs and 236 indels in GRCh38. This represents a significant increase in T2T‐CHM13 relative to GRCh38. These findings indicate a substantially higher proportion of variants with minimal read support in T2T‐CHM13, suggesting that although the gapless assembly offers a more complete genome, it also introduces more regions that are challenging for alignment and variant calling. Therefore, extra caution is warranted when using T2T‐CHM13‐based truth sets for supervised model training.

### 2.3 Analysis of the pretrained variants on T2T‐CHM13 reference

As shown in Figure 1, leveraging the GIAB benchmark variant set for the HG002 sample aligned to the T2T‐CHM13 reference, we assessed the comparative performance of the two assemblies. The T2T‐CHM13 reference demonstrated superior comprehensiveness, covering 90.61% of the benchmark regions compared to 82.32% for GRCh38. This enhanced completeness facilitated the discovery of a greater number of true variants, including SNPs and indels, and improved the genotyping of multi‐allelic sites. Consequently, the benchmark set for T2T‐CHM13 incorporates 29% more genomically challenging regions—as defined by the GIAB stratification—than the GRCh38 set. This increase is particularly pronounced in contexts such as low‐mappability areas, tandem repeats, and satellite regions, which are more accurately represented in the complete T2T‐CHM13 assembly.

**FIGURE 1:**
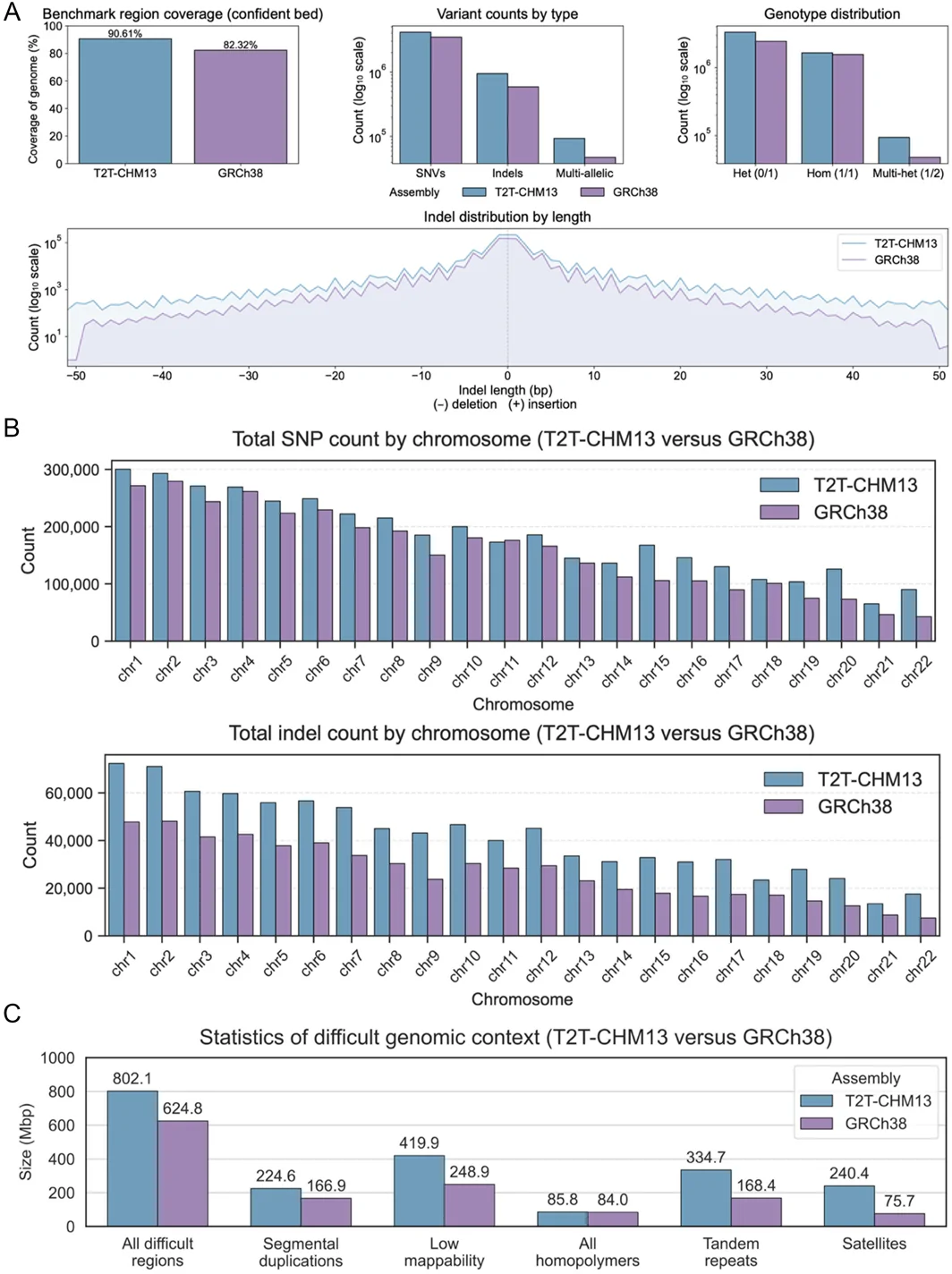
Comparison of variant counts and difficult genomic contexts between T2T‐CHM13 and GRCh38. (A) Benchmark region coverage, variant counts by type, genotype distribution, and indel distribution by length. The *y*‐axes for variant counts, genotype distribution, and indel distribution by length are shown on a log_10_ scale. Negative indel lengths indicate deletions, whereas positive indel lengths indicate insertions. (B) Total SNP and indel counts by chromosome in T2T‐CHM13 and GRCh38. (C) Statistics of difficult genomic contexts in T2T‐CHM13 and GRCh38. chr, chromosome; Het, heterozygous genotype; Hom, homozygous genotype; Multi‐het, multiallelic heterozygous genotype; SNP, single‐nucleotide polymorphism.

### 2.4 Performance on ONT R10.4.1 5 kHz T2T‐CHM13 datasets

We evaluated the performance of our models on ONT data using the HG002 sample across various sequencing coverages ranging from 10× to 50× on chromosome 20. The results are summarized in Figures 2, 3, 4, and Supporting Information S1: Table S3. Our analysis revealed that the mixed‐truth model demonstrated the most consistent overall performance across coverage levels, with its clearest advantage observed at lower sequencing depths, where coverage is limited. Notably, at 10× coverage, the mixed‐truth model achieved an SNP F1‐score of 96.32%. This performance is remarkably resilient, outperforming the GRCh38‐specific baseline, which achieved 95.84% in 10× HG002 T2T‐CHM13 dataset. This margin highlights the mixed‐truth model’s ability to maintain high precision and sensitivity even when read support is less abundant, suggesting that the integration of multi‐reference training data effectively enhances the model’s inference capability in challenging low‐coverage scenarios. As sequencing depth increased from 10× to 40×, the performance gap naturally narrowed as coverage saturation was reached, yet the mixed‐truth model maintained its lead. It achieved an average F1‐score of 99.25% for SNPs and 71.63% for indels, consistently surpassing the GRCh38‐specific baseline (99.10% for SNPs and 70.16% for indels). The observation that the mixed‐truth model remained competitive across the full coverage spectrum supports its use as a stable compromise between reference‐specific training schemes.

**FIGURE 2:**
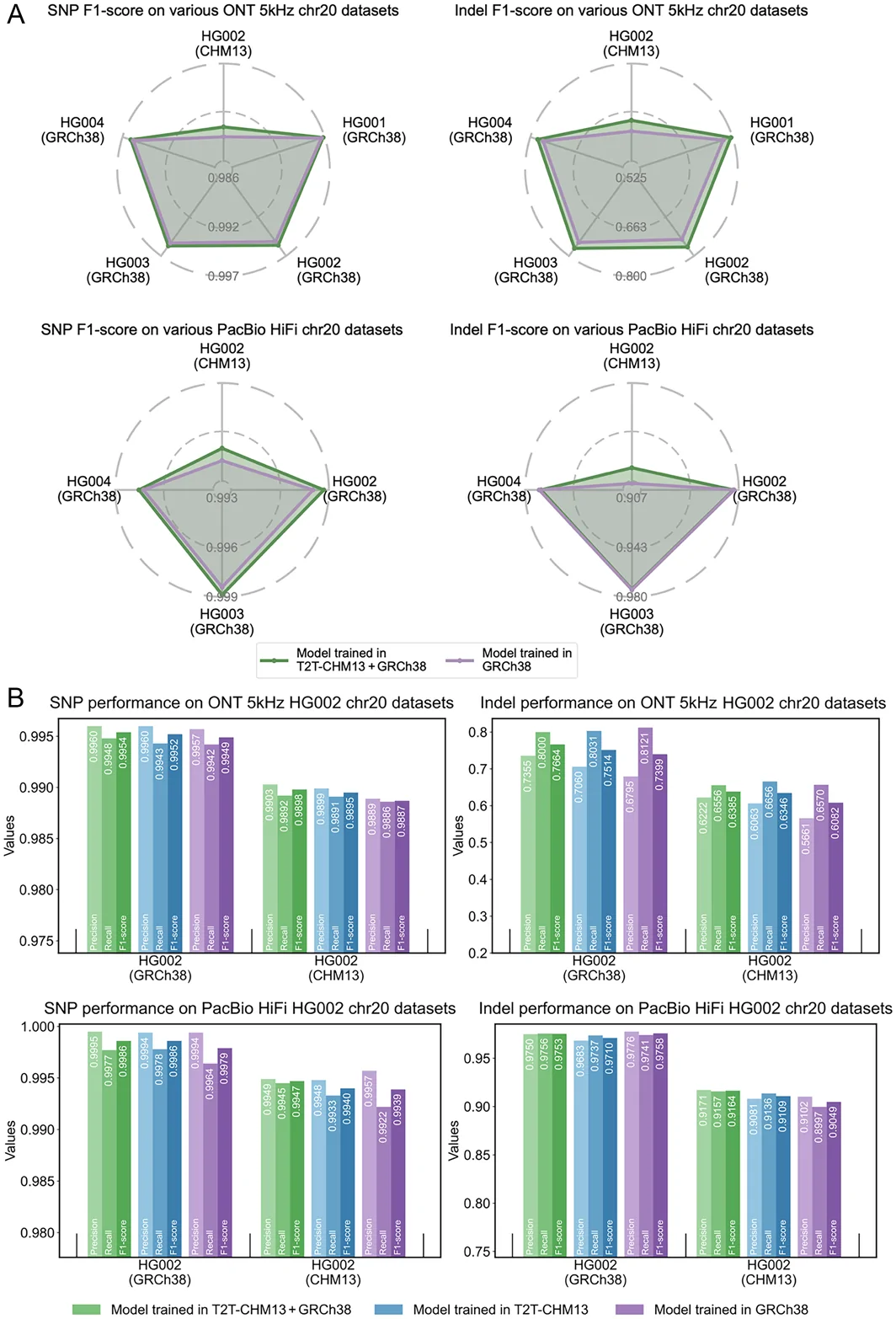
Performance comparison of variant calling models on chr20 datasets. (A) Radar plots showing SNP and indel F1‐scores on ONT 5 kHz and PacBio HiFi chr20 datasets from different samples and reference genomes. (B) Precision, recall, and F1‐score of SNP and indel calling on ONT 5 kHz and PacBio HiFi HG002 chr20 datasets using models trained in T2T‐CHM13 + GRCh38, T2T‐CHM13, or GRCh38. chr, chromosome; GRCh38, Genome Reference Consortium Human Build 38; HG, human genome sample; HiFi, high‐fidelity; ONT, Oxford Nanopore Technologies; SNP, single‐nucleotide polymorphism; indel, insertion and deletion; T2T‐CHM13, telomere‐to‐telomere CHM13 reference genome.

**FIGURE 3:**
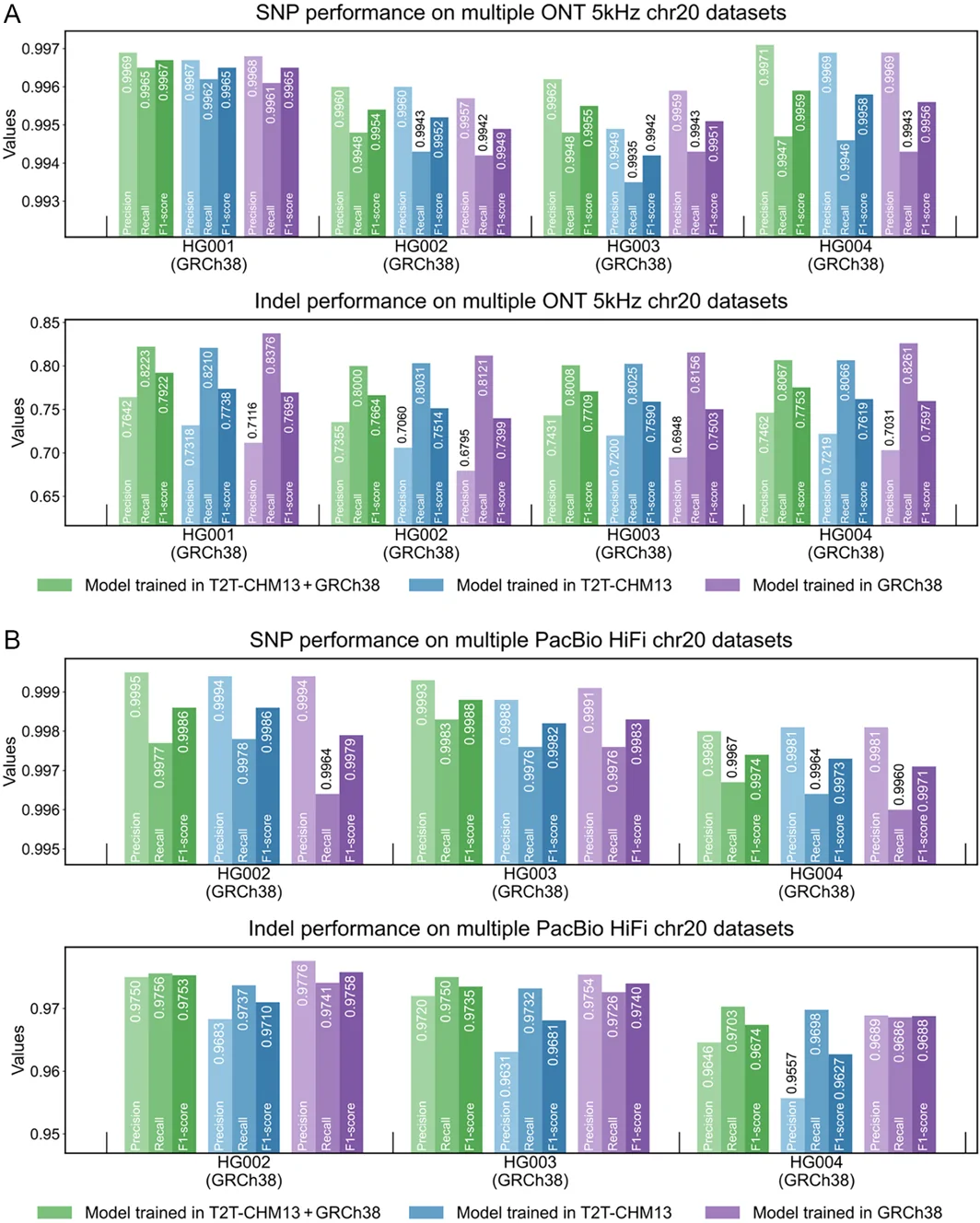
Evaluation of model performance across multiple GRCh38 chr20 datasets. (A) Precision, recall, and F1‐score of SNP and indel calling on ONT 5 kHz chr20 datasets from HG001, HG002, HG003, and HG004. (B) Precision, recall, and F1‐score of SNP and indel calling on PacBio HiFi chr20 datasets from HG002, HG003, and HG004. Models trained on T2T‐CHM13 + GRCh38, T2T‐CHM13, or GRCh38 were compared. chr, chromosome; GRCh38, Genome Reference Consortium Human Build 38; HG, human genome sample; HiFi, high‐fidelity; indel, insertion and deletion; ONT, Oxford Nanopore Technologies; SNP, single‐nucleotide polymorphism; T2T‐CHM13, telomere‐to‐telomere CHM13 reference genome.

**FIGURE 4:**
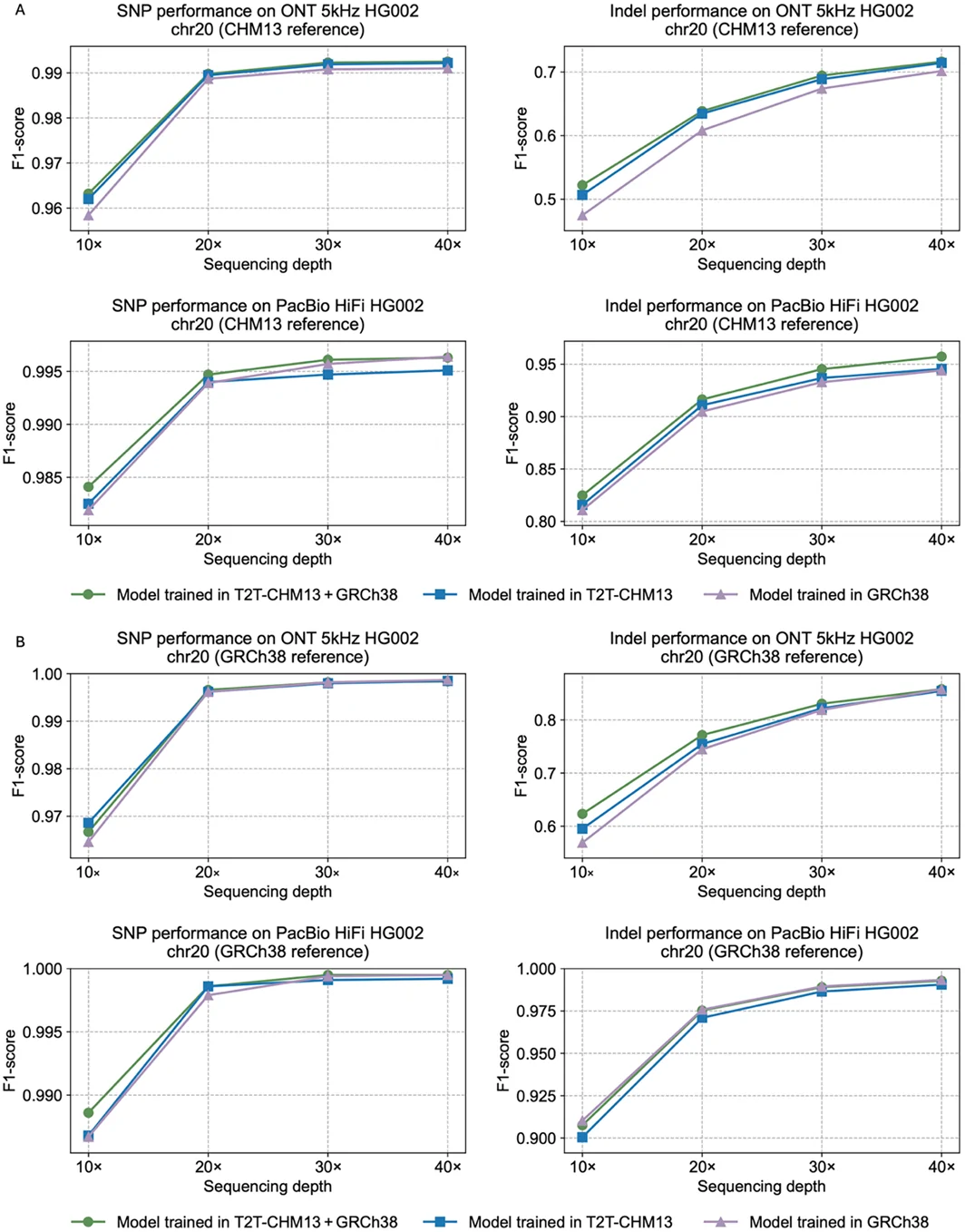
Evaluation of model performance across sequencing depths on HG002 chr20 datasets. (A) F1‐score of SNP and indel calling on ONT 5 kHz and PacBio HiFi HG002 chr20 data kHz and PacBio HiFi HG002 chr20 datasets aligned to the CHM13 reference at sequencing depths of 10×, 20×, 30×, and 40×. (B) F1‐score of SNP and indel calling on ONT 5 kHz and PacBio HiFi HG002 chr20 datasets aligned to the GRCh38 reference at sequencing depths of 10×, 20×, 30×, and 40×. Models trained on T2T‐CHM13 + GRCh38, T2T‐CHM13, or GRCh38 were compared. chr, chromosome; GRCh38, Genome Reference Consortium Human Build 38; HiFi, high‐fidelity; indel, insertion and deletion; ONT, Oxford Nanopore Technologies; SNP, single‐nucleotide polymorphism; T2T‐CHM13, telomere‐to‐telomere CHM13 reference genome.

In contrast, the model trained exclusively on T2T‐CHM13 (without mixing) failed to achieve comparable stability. The T2T‐CHM13‐specific model yielded an SNP F1‐score of only 96.20% at 10× coverage, which is 0.12% lower than the mixed‐truth model. Although its performance is comparable with the T2T‐CHM13 reference, its performance varied for the GRCh38 reference, with the mixed‐truth model outperforming the CHM13‐specific model by 0.47% in F1‐score (91.20% vs. 90.73%) on HG002 chr20 (10×). These comparisons support the practical utility of the mixed‐truth strategy: by combining the mature high‐confidence variant definitions available for GRCh38 with the expanded genomic context of T2T‐CHM13, the mixed model provides a balanced training scheme that improves robustness without strongly sacrificing performance on either reference. Moreover, to validate the source of these gains, we stratified performance into shared versus T2T‐specific regions—the latter enriched for complex segmental duplications and repeats. As detailed in Supporting Information S1: Table S6, the mixed‐truth model achieved robust improvements in these recalcitrant T2T‐specific intervals while preserving high precision in shared regions, confirming its ability to leverage expanded genomic context without compromising baseline accuracy.

### 2.5 Performance on different GRCh38 GIAB samples

In addition to the comparative analysis of the T2T‐CHM13 assembly, we further evaluated the generalizability of the models using the GIAB Ashkenazi Trio (HG002, HG003, HG004) and HG001 datasets, for which the variant calls have been fully validated against the GRCh38 reference genome. We compared the performance of our three pretrained models—namely, the mixed‐truth model, the CHM13‐specific model, and the GRCh38‐specific model—on chromosome 20 in all samples, which was excluded from the training data for all models to ensure an unbiased assessment. The mixed model demonstrated superior performance over the single‐reference models on the ONT platform across multiple samples and coverages. As shown in Figures 4 and 5 and Supporting Information S1: Table S2, the mixed‐truth model achieved generally leading performance across GIAB samples and reference genomes, particularly for overall and indel F1‐scores. For instance, in the HG003 data, the mixed‐truth model outperformed both the GRCh38‐specific and the CHM13‐specific models at 10× and 20× coverages, while maintaining highly competitive performance at higher coverages (exceeding 20×).

**FIGURE 5:**
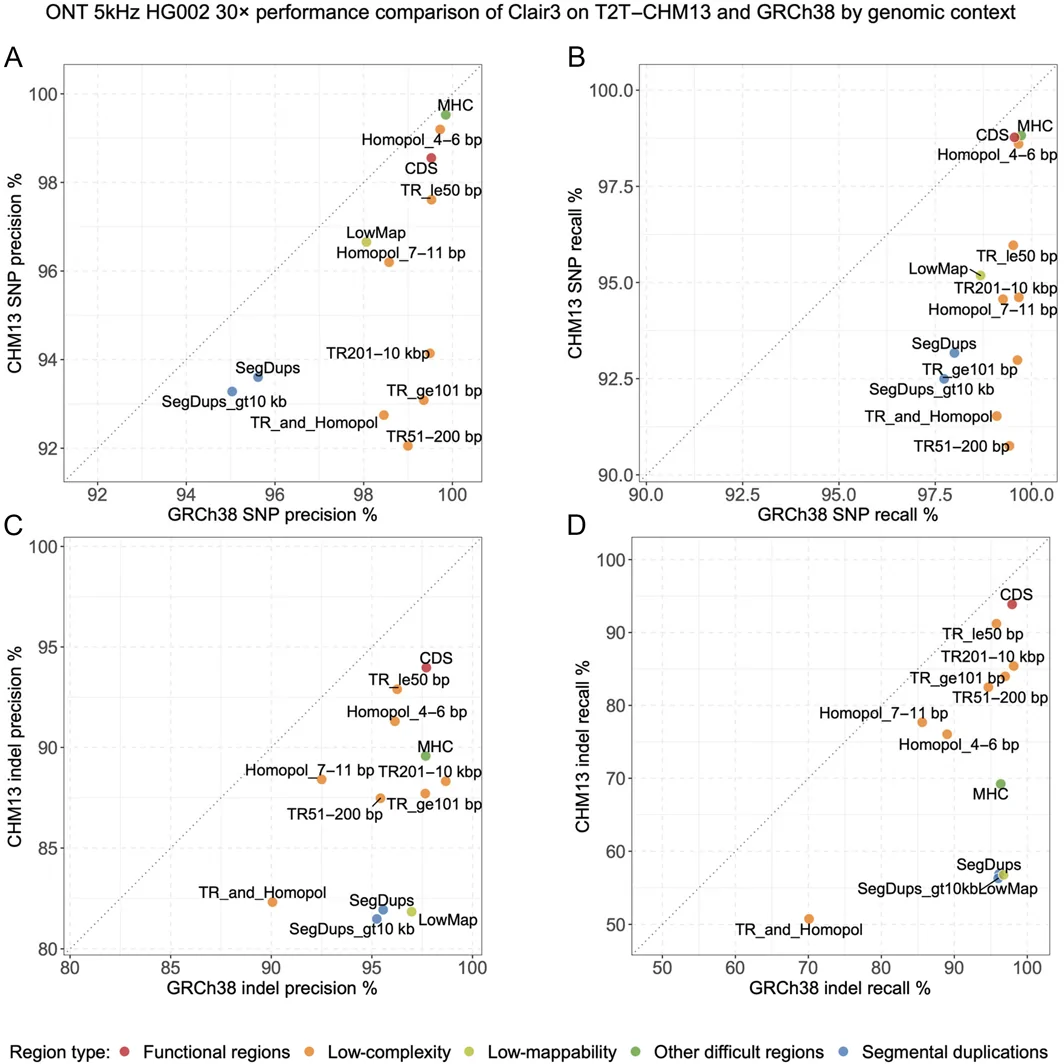
Performance comparison of Clair3 on T2T‐CHM13 and GRCh38 by genomic context using ONT 5 kHz HG002 30× chr20 data. (A) SNP precision. (B) SNP recall. (C) Indel precision. (D) Indel recall. Each point represents a genomic context or region type, and colors indicate region categories, including functional regions, low‐complexity regions, low‐mappability regions, other difficult regions, and segmental duplications. The *x*‐axis shows the performance obtained using GRCh38 as the reference, and the *y*‐axis shows the performance obtained using T2T‐CHM13 as the reference. The diagonal dashed line represents equal performance between the two references. Points above the diagonal indicate better performance with T2T‐CHM13, whereas points below the diagonal indicate better performance with GRCh38. CDS, coding sequence; chr, chromosome; GRCh38, Genome Reference Consortium Human Build 38; indel, insertion and deletion; MHC, major histocompatibility complex; ONT, Oxford Nanopore Technologies; SNP, single‐nucleotide polymorphism; T2T‐CHM13, telomere‐to‐telomere CHM13 reference genome; TR, tandem repeats.

Specifically, the mixed‐truth model achieved an overall F1‐score of 91.20% at 10× coverage and 96.43% at 20× coverage on HG002. In comparison, the GRCh38‐specific model achieved F1‐scores of 89.63% and 95.86% at the same coverages, representing a relative deficit of 1.57% and 0.57%, respectively. The CHM13‐specific model showed a minor performance gap, with F1‐scores of 90.73% and 96.09% at 10× and 20×, which are 0.47% and 0.34% lower than the mixed‐truth model, respectively. This performance advantage was more evident for the more challenging indel detection. At 20× coverage under the GRCh38 reference, the mixed‐truth model achieved an indel F1‐score of 77.16%, exceeding the GRCh38‐specific and CHM13‐specific models by 2.67% and 1.69%, respectively. At higher coverages, the mixed‐truth model remained slightly ahead in indel F1‐score, although the margin narrowed, particularly at 40× coverage. These results on an independent, fully‐validated GRCh38 truth set confirm the enhanced robustness of the mixed‐model approach. Its ability to outperform even the native GRCh38‐specific model on its home reference genome underscores the strategy’s effectiveness in learning a more generalized variant representation, which translates into improved accuracy and generalizability across different genomic contexts.

### 2.6 Performance analysis on different sequencing platforms

To assess the generalizability of our approach across sequencing technologies, we evaluated model performance on PacBio Revio HiFi data using the GIAB HG002 sample at 10×–40× coverage. As shown in Figure 4 and Supporting Information S1: Tables S4 and S5, the mixed model consistently outperformed the GRCh38‐specific and CHM13‐specific model across all coverages and variant types. In the HG002 sample at 10× coverage, the mixed model achieved an SNP F1‐score of 98.41% and an indel F1‐score of 82.48% on the T2T‐CHM13 reference. This represented a clear improvement over the GRCh38‐specific model, which attained 98.19% for SNPs and 81.08% for indels on the same reference. The CHM13 model trailed slightly with 98.25% for SNPs and 81.58% for indels. This pattern persisted at lower coverages, reinforcing the mixed model’s robustness: at 20× coverage, it maintained an SNP F1‐score of 99.47%, compared to 99.39% for the GRCh38‐specific model and 99.40% for the CHM13‐specific model. The performance gap was more pronounced in indel detection. Across PacBio HG002 downsampled datasets, the mixed‐truth model showed a consistent indel F1‐score advantage under the T2T‐CHM13 reference, exceeding those of the GRCh38‐specific and CHM13‐specific models by averages of 1.28% and 0.87%, respectively. Under the GRCh38 reference, the mixed‐truth model achieved indel F1‐scores comparable to the GRCh38‐specific model, with differences within 0.27%, and consistently outperformed the CHM13‐specific model by 0.23%–0.71%. This suggests that integrating variant representations from both references enhances sensitivity and precision in structurally variant‐rich regions, even when evaluating against the more complete—and thus more challenging—T2T‐CHM13 assembly.

To further validate platform consistency, we extended the benchmark to the Ashkenazi Trio (HG002–HG004) on GRCh38 reference. The mixed model delivered consistent results, achieving average overall F1‐scores of 99.47% and outperforming the GRCh38‐specific (99.47% vs. 99.44%) and CHM13‐specific (99.47% vs. 99.38%). These results confirm that the mixed‐model strategy provides a stable performance gain irrespective of the reference genome or sequencing technology used. Taken together, findings from both PacBio and ONT platforms affirm that a unified variant representation combining GRCh38 and CHM13 truth sets yields more accurate and robust variant calling than reliance on a single reference. This approach effectively harnesses the high‐confidence regions of GRCh38 while capitalizing on the expanded sequence context of T2T‐CHM13, establishing a more generalized and reliable framework for long‐read variant detection.

### 2.7 Performance analysis by different genomic contexts

The T2T‐CHM13 reference assembly has resolved a significant portion of the genome previously missing or incomplete in GRCh38, incorporating more complex genomic regions such as segmental duplications, low‐mappability regions, and satellite repeats. To quantitatively assess the variant calling performance in these challenging areas, we leveraged the GIAB benchmark stratification sets (v3.6 for GRCh38 and T2T‐CHM13) to evaluate our models on the HG002 sample. Our analysis focused on several categories defined by GIAB, including “low‐complexity,” “segmental duplications,” “low‐mappability,” “functional regions,” and “other difficult regions.” The results, illustrated in Figure 5, reveal a consistent trend: variant calling performance within these challenging contexts was systematically lower on the T2T‐CHM13 assembly compared to that on GRCh38.

This performance gap underscores the inherent difficulties introduced by the more complete but structurally complex landscape of CHM13. For example, in segmental duplications—a region type significantly expanded in CHM13—the F1‐score for indel detection was approximately 4.5% lower than in the corresponding GRCh38 regions. Furthermore, the absolute performance metrics in these difficult regions remained substantially lower than the whole‐genome averages. The mixed‐truth model achieved an overall F1‐score of 86.93% in challenging regions on T2T‐CHM13, compared to 93.86% for the whole genome. A similar disparity was observed for GRCh38 (85.70% vs. 91.92%), confirming that these contexts are universally difficult, but the challenge is exacerbated in the complete T2T‐CHM13. These findings indicate that while the T2T‐CHM13 reference provides a more comprehensive genomic framework, it also presents a greater challenge for accurate variant identification within its most complex regions where the GRCh38 has not covered.

## 3 DISCUSSION

The completion of the T2T‐CHM13 assembly represents a pivotal advance in genomics, providing the first truly complete, telomere‐to‐telomere reference genome. Our study systematically evaluates the impact of this transition on genetic variant detection using long‐read sequencing data. We demonstrate that while T2T‐CHM13 resolves persistent limitations of the GRCh38 reference, it also introduces new computational challenges—particularly in newly accessible complex genomic regions—demanding updated approaches for training and benchmarking variant calling methods.

The principal advantage of T2T‐CHM13 is its resolution of systemic gaps and misassemblies that have long characterized GRCh38, especially within structurally complex regions such as segmental duplications, pericentromeric zones, and satellite repeats [26]. By providing a more accurate and continuous genomic coordinate system, T2T‐CHM13 significantly improves read mapping fidelity. This reduces reference‐based alignment artifacts that previously led to both false‐positive variant calls and the omission of true variation in these difficult regions. As a result, T2T‐CHM13 enables more comprehensive and accurate variant discovery, fully leveraging the capabilities of long‐read sequencing technologies in previously inaccessible parts of the genome.

However, our analysis also revealed that the completeness of the T2T‐CHM13 reference introduces a new set of challenges. Although it provides a more comprehensive genomic landscape, it also incorporates complex genomic regions—such as satellite DNA and segmental duplications—that are absent or poorly represented in GRCh38. Variant calling in these newly accessible regions remains challenging, as evidenced by lower accuracy metrics, highlighting a critical area for future algorithm development. Consequently, to fully realize the potential of complete genome assemblies, the field must evolve its benchmarking standards. A key next step will be the development of expanded, high‐confidence variant sets that span these complex genomic territories, enabling the creation of methods capable of accurate variant discovery across the entire genome.

We note, however, that the benefit of mixed‐reference training should not be interpreted solely by the magnitude of the average genome‐wide F1‐score improvement. In several benchmarks, the observed gains were modest. We therefore view the mixed‐reference framework primarily as a practical transition strategy rather than a universally superior solution in all settings. Its main advantage is that it reduces dependence on a single reference‐specific truth space and provides more stable performance across references, sequencing platforms, and coverage regimes. This comes at the cost of additional truth curation and training complexity, and such complexity may not always be justified for applications that target only one mature reference assembly. Nevertheless, during the current transition from GRCh38 to T2T‐CHM13, we believe this trade‐off is reasonable because it offers a way to combine the maturity of existing GRCh38 benchmarks with the broader genomic representation afforded by T2T‐CHM13.

## 4 CONCLUSION

In this study, we introduced a novel strategy that integrates variant calls from both the T2T‐CHM13 and GRCh38 reference genomes to enhance variant calling performance. By combining the well‐curated, high‐confidence benchmarks from GRCh38 with the expanded and more complete genomic context of T2T‐CHM13, our hybrid training regimen successfully leverages the complementary strengths of both references. The mixed model demonstrated consistent superior performance across multiple sequencing technologies (ONT and PacBio), a range of coverage depths, and diverse sample sets, underscoring its robustness. Crucially, the mixed model surpassed the GRCh38‐exclusive baseline even when evaluated on the GRCh38 reference itself, indicating that the inclusion of T2T‐CHM13 confers benefits that go beyond standard dataset scaling. These gains transcend simple data augmentation, stemming instead from the information diversity provided by T2T‐CHM13. By incorporating complex contexts, the model acquires a robust reference‐independent representation that effectively decouples true biological signals from alignment artifacts.

## 5 MATERIALS AND METHODS

### 5.1 Overview of the overall evaluation metrics workflow

The evaluation of our method incorporates a comprehensive benchmarking strategy leveraging both the GRCh38 and T2T‐CHM13 reference assemblies and their corresponding high‐confidence truth sets. We adopted the core training workflow initially developed for the T2T‐CHM13 assembly across multiple experimental settings to systematically assess the impact of these distinct truth sets. As shown in Figure 6, to facilitate comprehensive benchmarking, we implemented three distinct training strategies to thoroughly characterize the performance of our model against different reference assemblies and truth sets. The GRCh38‐specific model served as a baseline, representing performance when trained exclusively on the long‐established GIAB high‐confidence calls based on the GRCh38 assembly. Conversely, the CHM13‐specific model (model trained in T2T‐CHM13 reference assembly) was developed to assess the inherent characteristics and performance gains achieved when training solely on the high‐quality relatively novel T2T‐CHM13 truth set, which offers a more complete representation of highly complex genomic regions. Finally, the mixed‐truth model was designed to investigate the feasibility of combining these two distinct truth spaces—GRCh38 and T2T‐CHM13—to determine if a model trained on diverse and complementary data could achieve a robust, generalized performance that leverages the strengths of both assemblies.

**FIGURE 6:**
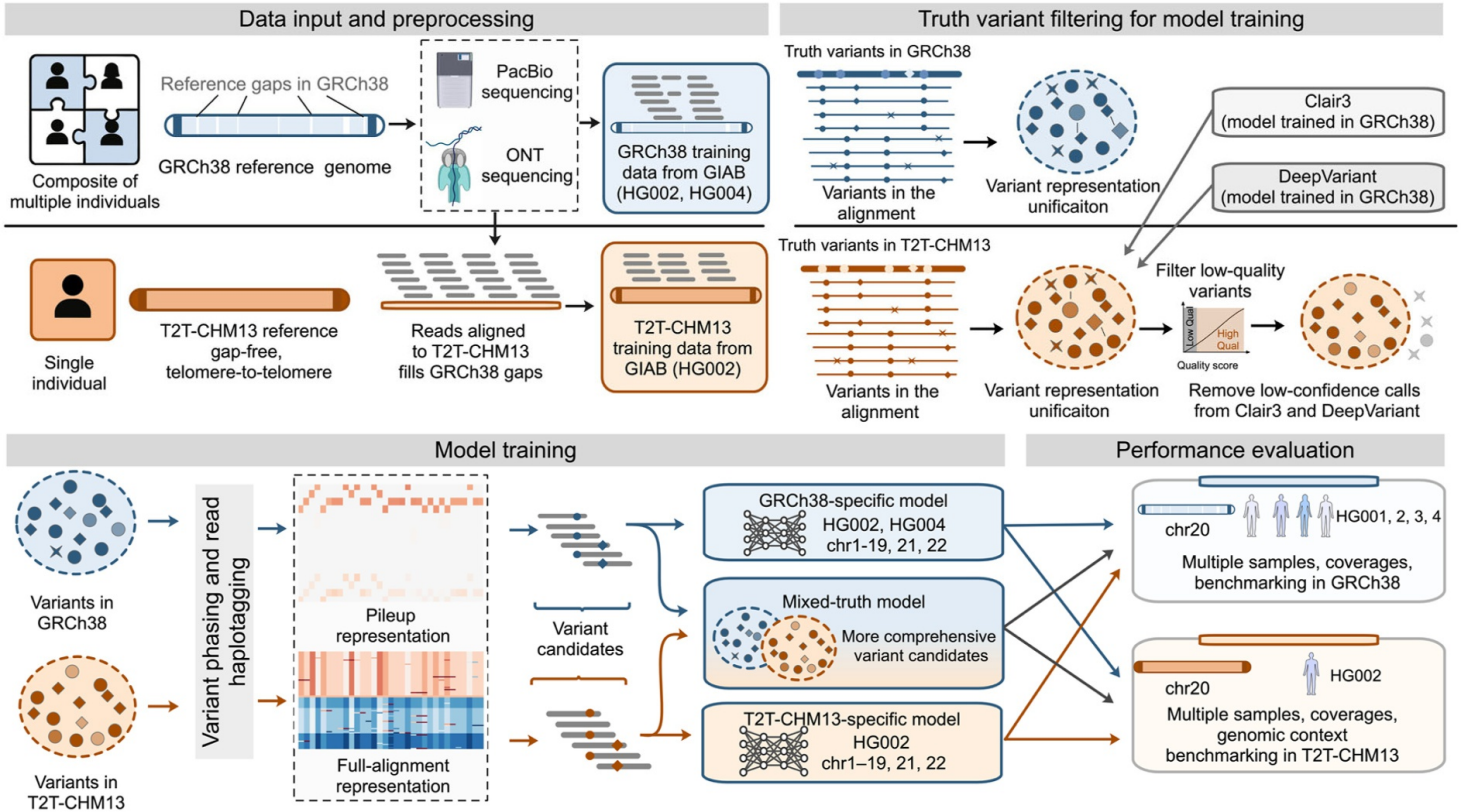
Workflow for training and evaluating reference‐specific and mixed‐truth variant calling models. PacBio HiFi and ONT sequencing data from GIAB samples were aligned to GRCh38 or T2T‐CHM13 during data preprocessing. Truth variants were extracted from the alignments, unified into a common variant representation, and filtered to remove low‐quality variants and low‐confidence calls from Clair3 and DeepVariant. Variant candidates were then generated through variant phasing, read haplotagging, pileup representation, and full‐alignment representation. Three model settings were trained: a GRCh38‐specific model, a T2T‐CHM13‐specific model, and a mixed‐truth model combining truth variants from both references. Model performance was evaluated on chromosome 20 across multiple samples, sequencing coverages, and genomic contexts using GRCh38‐and T2T‐CHM13‐based benchmarks. GIAB, Genome in a Bottle; GRCh38, Genome Reference Consortium Human Build 38; HiFi, high‐fidelity; ONT, Oxford Nanopore Technologies; T2T‐CHM13, telomere‐to‐telomere CHM13 reference genome.

For performance evaluation, our models were rigorously benchmarked using several key datasets: the WGS data for HG003, chromosome 20 data for HG002, and a comprehensive panel of chromosome 20 data spanning the GIAB samples HG001–HG004. The HG003 was used for WGS benchmarking, as our training model has excluded the HG003 dataset as a hold‐out dataset. This approach ensures a thorough assessment of model generalization and robustness across different individuals and data types. In the subsequent section, to provide essential context for our performance results, we will first present a brief comparison of the variant distribution within the GRCh38 and T2T‐CHM13 high‐confidence truth sets. This comparison will then be followed by a detailed overview of the CHM13‐specific training workflow that we employed.

### 5.2 Building well‐characterized training truths for model training on T2T‐CHM13 reference assembly

To establish high‐confidence training datasets for the T2T‐CHM13 model, we adopted a workflow analogous to that used for GRCh38, while implementing additional measures to enhance the reliability of the training labels. Given that the truth variants in T2T‐CHM13 assembly remain a draft version, we introduced several validations to ensure the accuracy of the training truths.

First, variant representations were unified using Repun, a method designed to align variant records in a truth VCF with the supporting evidence from sequencing alignments without performing variant calling. This step ensures that training labels are consistent with the underlying sequencing data, thereby improving the suitability of the data for deep learning‐based variant caller training and enabling direct evaluation of alignment quality. Additionally, to minimize interference from low‐frequency variants during model training, we excluded all variant candidates with a variant allele frequency below 0.05, regardless of whether they were designated as variant or nonvariant sites. This filtering step reduces noise and enhances the robustness of the training set. To further prevent the inclusion of T2T‐CHM13‐specific artifacts in the training data, we applied an initial variant calling step using both Clair3 (v1.2.0) and DeepVariant (v1.10) with their respective GRCh38 models. We then excluded the bottom 5% of low‐quality SNPs and indels, as informed by Repun’s output. Those variants would not be used as variant category or a sequencing artifact category. This filtering is critical to prune candidates in complex T2T‐CHM13 regions that exhibit anomalous out‐of‐distribution features—which are sequence and alignment patterns that differ from those represented in the training data—driven by local alignment noise. This approach ensures that the model is not exposed to potential platform‐ or assembly‐specific inaccuracies, observed in T2T‐CHM13 reference compared to GRCh38‐based benchmarks.

In the benchmarking phase, these filtering criteria were intentionally relaxed to evaluate the factors contributing to false negatives and false positives. This allows an unbiased assessment of variant calling performance on the T2T‐CHM13 dataset and helps identify underlying sources of error.

### 5.3 Construction of haplotype‐aware training data in T2T‐CHM13

Our model training pipeline involved three key stages, beginning with sample‐aligned sequencing data as input. From these alignments, we extracted candidate genomic loci, which were systematically classified as either true variants or artifacts. True variants were further delineated into homozygous, heterozygous, and multi‐allelic sites to inform the model training. A critical step was the integration of haplotype information. We first phased the heterozygous variants from our high‐confidence truth set directly using the sample alignments. These phased variants were then used to assign haplotype tags to the sequencing reads, a process known as haplotagging. This approach provides a significant advantage over using variants called de novo: by leveraging curated truths for phasing, we ensure the long‐range haplotype information is anchored to high‐confidence positions, thereby minimizing errors. This results in a more robust foundation for model training, which is particularly crucial for long‐read data when accurate prediction across variable coverage and allele frequencies is required.

Subsequently, the haplotagged alignments were processed by Repun to identify and resolve inconsistencies in variant representation. The inclusion of haplotype information here significantly reduces computational complexity by constraining the possible allelic combinations. Finally, these processed haplotagged alignments were subjected to downsampling to augment the training data and then used to train a full‐alignment deep learning model. For the mixed‐truth model, we directly aggregated the complete training datasets from both GRCh38 and T2T‐CHM13. Because the total number of examples from each assembly was comparable, this full integration naturally ensured balanced exposure to different variant types.

### 5.4 Benchmarking datasets and alignment configurations

In this study, we evaluated the performance of the variant calling methods using long‐read sequencing data from both ONT and PacBio platforms. For ONT, we utilized data for the GIAB samples HG001–HG004, which were generated by Nanopore EPI2ME Labs using the latest chemistry (R10.4.1, 5 kHz) and the SQK‐LSK114 sequencing kit (ONT, Oxford, UK). For the PacBio data, we used the sequencing data provided by the PacBio research team, which were generated on the Revio system using the SMRT prep kit 3.0 (Pacific Biosciences of California, Inc., Menlo Park, CA, USA). All datasets were initially aligned to the GRCh38 reference genome. For our analysis, we realigned these reads to the T2T‐CHM13 reference genome using the platform‐specific aligners minimap2 [27] for ONT data and pbmm2 (v26.1.0) for PacBio data alignment. All commands and parameters used for sequence alignment are provided in the Supplementary Information—command lines used section.

We selected minimap2 as the ONT aligner because it is widely used in long‐read small‐variant calling workflows and provides a practical baseline that is compatible with established Clair3 benchmarking settings. We note, however, that aligner choice may have a stronger impact in repeat‐rich and low‐mappability regions, particularly in the newly resolved sequence context of T2T‐CHM13. In this context, repeat‐aware aligners such as Winnowmap may influence absolute performance and possibly alter the magnitude of the gains observed here.

### 5.5 Performance evaluation across various challenging genomic contexts

To assess variant calling performance across different genomic contexts, we employed stratification bed files obtained from the GIAB Consortium. These files categorize the genome into distinct functional and evolutionary regions based on standardized criteria. We utilized the GIAB stratification [28] version 3.6 for GRCh38 and T2T‐CHM13 reference genome. Our analysis specifically focused on categories recognized as challenging for both short‐read and long‐read sequencing technologies, such as segmental duplications, low‐mappability, low‐complexity, and other complex genomic regions.

It is important to note that these stratifications are defined genome‐wide and are not tailored to sample‐specific high‐confidence regions. Therefore, for a precise performance evaluation, we restricted our analysis to variants that fell within both the genomic stratification categories and the sample‐specific high‐confidence regions. Performance metrics, including precision and recall, were calculated by comparing the called variants, missed variants (false negatives), and artifactual calls (false positives) within these intersecting regions. For the T2T‐CHM13‐based evaluation, a comparative analysis was limited by the availability of only a single high‐confidence truth set (the HG002 sample). To enable a controlled comparison, we adopted a specific training regimen: models were trained on chromosomes 1–19 and 21–22, while chromosome 20 was held out as an independent benchmark set. This design allowed us to benchmark performance on the T2T‐CHM13 assembly while simultaneously evaluating the model’s ability to generalize to a chromosome unseen during training, thus isolating the impact of incorporating T2T‐CHM13‐specific variants in the training data.

### 5.6 Benchmarking metrics

We used GIAB samples for benchmarking. For the variant calling performance, we used hap.py [29] to compare the called variants against the true variants and used Clair3’s “GetOverallMetrics” module to generate three metrics (“precision”, “recall” and “F1‐score”) for five categories (“overall,” “SNP,” “indel,” “insertion” and “deletion”).

### 5.7 Computational performance

Clair3 was implemented with Python (3.10.10) and TensorFlow (2.21.0) and leveraged C in feature generation. The memory consumption is capped at 500 MB per process. Model training on T2T‐CHM13 and GRCh38 references requires a high‐end graphics processing unit. We used Tensorflow to train models on an NVIDIA GeForce RTX™ 3090 and 4090 GPU.

### 5.8 Code availability

Clair3 is open source and available from the HKU‐BAL Clair3 repository on GitHub under the BSD 3‐Clause license. Training documentation for pileup and full‐alignment models is provided in the same repository. Pretrained model weights are available from the Clair3 model download page maintained by our group. Detailed access information is provided in the Supplementary Notes. Users can run the workflow seamlessly in one command with the input BAM file and reference file using Docker (engine version 25.0.3) or Bioconda [30] (conda 23.7.4).

## AUTHOR CONTRIBUTIONS

**Zhenxian Zheng**: Methodology; software; writing—original draft; writing—review and editing. **Minggao He**: Validation; formal analysis; writing—original draft; writing—review and editing. **Xian Yu**: Validation; formal analysis; writing—review and editing. **Lei Chen**: Validation; formal analysis; writing—review and editing. **Junzhe Li**: Formal analysis; writing—review and editing. **Jingcheng Zhang**: Writing—review and editing. **Ruibang Luo**: Conceptualization; methodology; supervision; writing—original draft; writing—review and editing.

## CONFLICT OF INTEREST STATEMENT

The authors declare no conflicts of interest.

## ETHICS STATEMENT

This study utilized publicly available, de‐identified genomic data from the Genome in a Bottle (GIAB) Consortium. Therefore, additional informed consent was not required.

## Supporting information

Supporting Information S1

## Data Availability

The (1) links to the reference genomes, truth variants, benchmarking materials, and (2) the benchmarked commands and parameters used in this study are available in the Supporting Information.
