# Supplementary material for: Telomere‐to‐telomere CHM13 reference reveals missing truth variants and improves deep learning‐based variant calling in long‐read sequencing data
Source: Quant Biol. 2026 Aug 18;14(4):e70051. doi: 10.1002/qub2.70051 (PMC13642155; doi:10.1002/qub2.70051)
Supplement: Supplementary file 1 [file QUB2-14-e70051-s001.pdf]

Supplementary Notes

Supplementary Tables

Supplementary Table 1. Performance analysis of the unified variants on HG002 using T2T-CHM13 and GRCh38 reference.

Platform	Dataset	Reference genome	Overall Precision	Overall Recall	Overall F1-score	SNP Precision	SNP Recall	SNP F1-score	INDEL Precision	INDEL Recall	Indel F1-score
ONT	HG002 WGS	T2T-CHM13	99.99%	99.21%	99.60%	99.99%	99.67%	99.83%	99.99%	97.30%	98.63%
		HG38	100.00%	99.95%	99.97%	100.00%	99.97%	99.98%	99.99%	99.80%	99.90%
PacBio		T2T-CHM13	99.91%	99.60%	99.76%	99.94%	99.74%	99.84%	99.79%	99.02%	99.41%
		HG38	100.00%	99.95%	99.97%	100.00%	99.95%	99.97%	100.00%	99.96%	99.98%

Supplementary Table 2. Performance comparison for different pre-trained models, evaluated across different ONT GIAB samples and reference genomes.

Platform	Reference	Dataset	Caller	Overall Precision	Overall Recall	Overall F1-score	SNP Precision	SNP Recall	SNP F1-score	INDEL Precision	INDEL Recall	Indel F1-score
ONT	HG38	HG001 chr20	The mixed-truth model	96.50%	97.46%	96.97%	99.69%	99.65%	99.67%	76.42%	82.23%	79.22%
			GRch38-specific model	95.45%	97.61%	96.52%	99.68%	99.61%	99.65%	71.16%	83.76%	76.95%
			CHM13-specific model	95.91%	97.41%	96.65%	99.67%	99.62%	99.65%	73.18%	82.10%	77.38%
		HG002 chr20	The mixed-truth model	95.70%	96.83%	96.26%	99.60%	99.48%	99.54%	73.55%	80.00%	76.64%
			GRch38-specific model	94.44%	96.94%	95.67%	99.57%	99.42%	99.49%	67.95%	81.21%	73.99%
			CHM13-specific model	95.10%	96.83%	95.95%	99.60%	99.43%	99.52%	70.60%	80.31%	75.14%
		HG003 chr20	The mixed-truth model	95.98%	96.93%	96.45%	99.62%	99.48%	99.55%	74.31%	80.08%	77.09%
			GRch38-specific model	94.93%	97.08%	95.99%	99.59%	99.43%	99.51%	69.48%	81.56%	75.03%
			CHM13-specific model	95.42%	96.84%	96.12%	99.49%	99.35%	99.42%	72.00%	80.25%	75.90%
		HG004 chr20	The mixed-truth model	96.05%	96.97%	96.51%	99.71%	99.47%	99.59%	74.62%	80.67%	77.53%
			GRch38-specific model	95.08%	97.19%	96.13%	99.69%	99.43%	99.56%	70.31%	82.61%	75.97%
			CHM13-specific model	95.56%	96.96%	96.26%	99.69%	99.46%	99.58%	72.19%	80.66%	76.19%
	T2T-CHM13	HG002 chr20	The mixed-truth model	91.28%	92.49%	91.88%	99.03%	98.92%	98.98%	62.22%	65.56%	63.85%
			GRch38-specific model	89.24%	92.46%	90.82%	98.89%	98.86%	98.87%	56.61%	65.70%	60.82%
			CHM13-specific model	90.65%	92.67%	91.65%	98.99%	98.91%	98.95%	60.63%	66.56%	63.46%

Supplementary Table 3. Performance evaluation of different models across multiple sequencing coverage on ONT HG002.

Platform	Reference	Dataset/ coverage	Caller	Overall Precision	Overall Recall	Overall F1-score	SNP Precision	SNP Recall	SNP F1-score	INDEL Precision	INDEL Recall	Indel F1-score
ONT	T2TCHM13	HG002 10x	The mixed-truth model	84.12%	89.10%	86.54%	96.52%	96.11%	96.32%	46.34%	59.79%	52.21%
			GRch38-specific model	80.37%	88.72%	84.34%	95.93%	95.74%	95.84%	39.58%	59.33%	47.48%
			CHM13-specific model	83.03%	88.88%	85.86%	96.58%	95.83%	96.20%	43.98%	59.80%	50.68%
		HG002 20x	The mixed-truth model	91.28%	92.49%	91.88%	99.03%	98.92%	98.98%	62.22%	65.56%	63.85%
			GRch38-specific model	89.24%	92.46%	90.82%	98.89%	98.86%	98.87%	56.61%	65.70%	60.82%
			CHM13-specific model	90.65%	92.67%	91.65%	98.99%	98.91%	98.95%	60.63%	66.56%	63.46%
		HG002 30x	The mixed-truth model	94.00%	92.98%	93.49%	99.39%	99.06%	99.23%	71.51%	67.51%	69.45%
			GRch38-specific model	92.52%	92.97%	92.75%	99.22%	98.94%	99.08%	66.83%	67.99%	67.40%
			CHM13-specific model	93.43%	93.10%	93.27%	99.30%	99.08%	99.19%	69.73%	68.05%	68.88%
		HG002 40x	The mixed-truth model	95.05%	93.03%	94.03%	99.50%	99.00%	99.25%	75.59%	68.06%	71.63%
			GRch38-specific model	93.82%	93.07%	93.44%	99.35%	98.84%	99.10%	71.47%	68.90%	70.16%
			CHM13-specific model	94.61%	93.21%	93.90%	99.42%	99.02%	99.22%	74.20%	68.86%	71.43%
	HG38	HG002 10x	The mixed-truth model	89.05%	93.46%	91.20%	96.73%	96.60%	96.67%	54.09%	73.50%	62.32%
			GRch38-specific model	86.05%	93.52%	89.63%	96.34%	96.58%	96.46%	46.18%	74.11%	56.90%
			CHM13-specific model	88.26%	93.33%	90.73%	97.15%	96.57%	96.86%	50.34%	72.82%	59.53%
		HG002 20x	The mixed-truth model	95.81%	97.06%	96.43%	99.70%	99.63%	99.66%	73.85%	80.77%	77.16%
			GRch38-specific model	94.56%	97.18%	95.86%	99.63%	99.61%	99.62%	68.37%	81.81%	74.49%
			CHM13-specific model	95.22%	96.98%	96.09%	99.68%	99.57%	99.62%	70.94%	80.61%	75.47%
		HG002 30x	The mixed-truth model	97.60%	97.44%	97.52%	99.85%	99.79%	99.82%	83.58%	82.55%	83.06%
			GRch38-specific model	96.99%	97.61%	97.30%	99.85%	99.80%	99.82%	80.13%	83.76%	81.90%
			CHM13-specific model	97.30%	97.44%	97.37%	99.83%	99.78%	99.80%	81.81%	82.60%	82.20%
		HG002 40x	The mixed-truth model	98.25%	97.65%	97.95%	99.85%	99.82%	99.84%	87.88%	83.83%	85.81%
			GRch38-specific model	98.00%	97.88%	97.94%	99.89%	99.85%	99.87%	86.22%	85.37%	85.79%
			CHM13-specific model	98.13%	97.67%	97.90%	99.87%	99.83%	99.85%	87.03%	83.96%	85.47%

Supplementary Table 4. Performance comparison for different pre-trained models, evaluated across different PacBio GIAB samples and reference genomes.

Platform	Reference	Dataset	Caller	Overall Precision	Overall Recall	Overall F1-score	SNP Precision	SNP Recall	SNP F1-score	INDEL Precision	INDEL Recall	Indel F1-score
PacBio	HG38	HG002 chr20	The mixed-truth model	99.61%	99.47%	99.54%	99.95%	99.77%	99.86%	97.50%	97.56%	97.53%
			GRch38-specific model	99.64%	99.34%	99.49%	99.94%	99.64%	99.79%	97.76%	97.41%	97.58%
			CHM13-specific model	99.50%	99.45%	99.48%	99.94%	99.78%	99.86%	96.83%	97.37%	97.10%
		HG003 chr20	The mixed-truth model	99.56%	99.52%	99.54%	99.93%	99.83%	99.88%	97.20%	97.50%	97.35%
			GRch38-specific model	99.59%	99.43%	99.51%	99.91%	99.76%	99.83%	97.54%	97.26%	97.40%
			CHM13-specific model	99.39%	99.44%	99.41%	99.88%	99.76%	99.82%	96.31%	97.32%	96.81%
		HG004 chr20	The mixed-truth model	99.34%	99.32%	99.33%	99.80%	99.67%	99.74%	96.46%	97.03%	96.74%
			GRch38-specific model	99.41%	99.24%	99.32%	99.81%	99.60%	99.71%	96.89%	96.86%	96.88%
			CHM13-specific model	99.22%	99.29%	99.26%	99.81%	99.64%	99.73%	95.57%	96.98%	96.27%
	T2T-CHM13	HG002 chr20	The mixed-truth model	97.90%	97.93%	97.92%	99.49%	99.45%	99.47%	91.71%	91.57%	91.64%
			GRch38-specific model	97.84%	97.43%	97.64%	99.57%	99.22%	99.39%	91.02%	89.97%	90.49%
			CHM13-specific model	97.70%	97.79%	97.75%	99.48%	99.33%	99.40%	90.81%	91.36%	91.09%

Supplementary Table 5. Performance evaluation of different models across multiple sequencing coverage on PacBio HG002.

Platform	Reference	Dataset	Caller	Overall Precision	Overall Recall	Overall F1-score	SNP Precision	SNP Recall	SNP F1-score	INDEL Precision	INDEL Recall	Indel F1-score
PacBio	T2T-CHM13	HG002 10x	The mixed-truth model	95.59%	94.93%	95.26%	98.85%	97.98%	98.41%	82.79%	82.17%	82.48%
			GRch38-specific model	95.95%	93.84%	94.88%	98.96%	97.44%	98.19%	83.53%	78.78%	81.08%
			CHM13-specific model	94.98%	94.84%	94.91%	98.68%	97.82%	98.25%	80.79%	82.38%	81.58%
		HG002 20x	The mixed-truth model	97.90%	97.93%	97.92%	99.49%	99.45%	99.47%	91.71%	91.57%	91.64%
			GRch38-specific model	97.84%	97.43%	97.64%	99.57%	99.22%	99.39%	91.02%	89.97%	90.49%
			CHM13-specific model	97.70%	97.79%	97.75%	99.48%	99.33%	99.40%	90.81%	91.36%	91.09%
		HG002 30x	The mixed-truth model	98.58%	98.62%	98.60%	99.60%	99.61%	99.61%	94.60%	94.45%	94.53%
			GRch38-specific model	98.35%	98.30%	98.33%	99.61%	99.54%	99.57%	93.47%	93.11%	93.29%
			CHM13-specific model	98.35%	98.29%	98.32%	99.59%	99.36%	99.47%	93.55%	93.82%	93.68%
		HG002 40x	The mixed-truth model	98.84%	98.87%	98.86%	99.63%	99.64%	99.63%	95.78%	95.67%	95.73%
			GRch38-specific model	98.60%	98.60%	98.60%	99.66%	99.61%	99.64%	94.45%	94.37%	94.41%
			CHM13-specific model	98.54%	98.51%	98.52%	99.58%	99.44%	99.51%	94.51%	94.62%	94.56%
	HG38	HG002 10x	The mixed-truth model	98.16%	97.31%	97.74%	99.38%	98.35%	98.86%	90.78%	90.73%	90.76%
			GRch38-specific model	98.48%	96.80%	97.63%	99.39%	97.97%	98.67%	92.75%	89.37%	91.03%
			CHM13-specific model	97.72%	97.23%	97.47%	99.17%	98.21%	98.68%	89.07%	91.05%	90.05%
		HG002 20x	The mixed-truth model	99.61%	99.47%	99.54%	99.95%	99.77%	99.86%	97.50%	97.56%	97.53%
			GRch38-specific model	99.64%	99.34%	99.49%	99.94%	99.64%	99.79%	97.76%	97.41%	97.58%
			CHM13-specific model	99.50%	99.45%	99.48%	99.94%	99.78%	99.86%	96.83%	97.37%	97.10%
		HG002 30x	The mixed-truth model	99.84%	99.77%	99.81%	99.99%	99.91%	99.95%	98.92%	98.91%	98.91%
			GRch38-specific model	99.84%	99.77%	99.81%	99.99%	99.90%	99.94%	98.91%	99.00%	98.95%
			CHM13-specific model	99.78%	99.70%	99.74%	99.98%	99.85%	99.91%	98.56%	98.75%	98.65%
		HG002 40x	The mixed-truth model	99.89%	99.83%	99.87%	99.99%	99.91%	99.95%	99.25%	99.32%	99.29%
			GRch38-specific model	99.90%	99.83%	99.87%	99.99%	99.92%	99.95%	99.34%	99.34%	99.34%
			CHM13-specific model	99.86%	99.74%	99.80%	99.99%	99.85%	99.92%	99.03%	99.09%	99.06%

Supplementary Table 6. Performance comparison T2T-specific high-confidence regions.

Dataset	Coverage	Caller	Type	TP	FP	FN	Precision	Recall	F1-score
ONT HG002 chr20 (Variant in CHM13's BED while not in GRCh38's BED)	10x	GRch38-specific model	SNP	5657	986	843	85.16%	87.03%	86.08%
		GRch38-specific model	INDEL	3059	7538	4243	28.87%	41.89%	34.18%
		CHM13-specific model	SNP	5648	930	823	85.86%	87.28%	86.57%
		CHM13-specific model	INDEL	3202	6504	4065	32.99%	44.06%	37.73%
		The mixed-truth model	SNP	5686	908	802	86.23%	87.64%	86.93%
		The mixed-truth model	INDEL	3191	6205	4105	33.96%	43.74%	38.23%
		DeepVariant	SNP	4905	437	1619	91.82%	75.18%	82.67%
		DeepVariant	INDEL	1522	885	5569	63.23%	21.46%	32.05%
	20x	GRch38-specific model	SNP	5874	586	565	90.93%	91.23%	91.08%
		GRch38-specific model	INDEL	3423	4414	3864	43.68%	46.97%	45.27%
		CHM13-specific model	SNP	5909	601	548	90.77%	91.51%	91.14%
		CHM13-specific model	INDEL	3589	4669	3710	43.46%	49.17%	46.14%
		The mixed-truth model	SNP	5970	627	509	90.50%	92.14%	91.31%
		The mixed-truth model	INDEL	3446	5386	3908	39.02%	46.86%	42.58%
		DeepVariant	SNP	5599	363	908	93.91%	86.05%	89.81%
		DeepVariant	INDEL	2273	1509	4894	60.10%	31.71%	41.52%
	30x	GRch38-specific model	SNP	5935	521	523	91.93%	91.90%	91.92%
		GRch38-specific model	INDEL	3585	4153	3812	46.33%	48.47%	47.37%
		CHM13-specific model	SNP	5872	479	582	92.46%	90.98%	91.71%
		CHM13-specific model	INDEL	3599	3597	3708	50.01%	49.25%	49.63%
		The mixed-truth model	SNP	5853	441	586	92.99%	90.90%	91.93%
		The mixed-truth model	INDEL	3507	3292	3803	51.58%	47.98%	49.71%
		DeepVariant	SNP	5744	383	755	93.75%	88.38%	90.99%
		DeepVariant	INDEL	2624	1796	4584	59.37%	36.40%	45.13%

Command line used

BAM subsampling

Samtools(v1.10)

```
samtools view -@ ${THREADS} -s 0.${RATIO} -b -o subsampled.bam ${BAM}
samtools index -@ ${THREADS} subsampled.bam
```

Coverage calculation

Mosdepth(v0.2.9)

```
mosdepth -t ${THREADS} -n -x --quantize 0:15:150: output ${BAM}
```

Running Clair3 (v1.2.0)

```
bash run_clair3.sh \
-b {INPUT_DIR}/sample.bam \
-f ${INPUT_DIR}/ref.fa \
-m ${MODEL_PATH} \
-t ${THREAD} \
-p ${PLATFORM} \
-o ${OUTPUT_DIR}
```

Benchmarking

hap.py (v0.3.12)

```
hap.py ${GIAB_BASELINE_VCF} output.vcf.gz \
 -o ${OUTPUT_DIR}/happy \
 -r ${REF} \
 -f ${GIAB_CONFIDENT_BED} \
 --threads ${THREADS} \
 --pass-only \
 --engine=vcfeval

# Overall insertion and deletion Precision, Recall, F1-Score
pypy3 ${REPUN} GetOverallMetrics \
 --happy_vcf_fn ${OUTPUT_DIR}/happy.vcf.gz \
 --output_fn happy.log
```

Data availability

Reference genomes

GRCh38 reference

ftp.ncbi.nlm.nih.gov/genomes/all/GCA/000/001/405/GCA_000001405.15_GRCh38/seqs_for_alignment_pipelines.ucsc_ids /GCA_000001405.15_GRCh38_no_alt_analysis_set.fna.gz

T2T-CHM13 reference

github.com/marbl/T2T-Browser

GIAB truth variants

HG001 (NA12878), GRCh38, v4.2.1

ftp-trace.ncbi.nlm.nih.gov/giab/ftp/release/NA12878_HG001/NISTv4.2.1/GRCh38/

HG002 (NA24385), GRCh38, v4.2.1

ftp-trace.ncbi.nlm.nih.gov/giab/ftp/release/AshkenazimTrio/HG002_NA24385_son/NISTv4.2.1/GRCh38/

HG003 (NA24149), GRCh38, v4.2.1

ftp-trace.ncbi.nlm.nih.gov/giab/ftp/release/AshkenazimTrio/HG003_NA24149_father/NISTv4.2.1/GRCh38/

HG004 (NA24143), GRCh38, v4.2.1

ftp-trace.ncbi.nlm.nih.gov/giab/ftp/release/AshkenazimTrio/HG004_NA24143_mother/NISTv4.2.1/GRCh38/

HG002 (NA24385), T2T-CHM13, v1.1

ftp-trace.ncbi.nlm.nih.gov/ReferenceSamples/giab/data/AshkenazimTrio/analysis/NIST_HG002_DraftBenchmark_defrabbV0.020-20250117/

Oxford Nanopore (ONT) sequencing data

ONT EPI2ME Labs HG001-4 R10.4.1 Q20+, GRCh38

labs.epi2me.io/giab-2023.05/

PacBio Sequencing Data

PacBio HG002 HiFi Revio, GRCh38

downloads.pacbcloud.com/public/revio/2022Q4/HG002-rep1/

PacBio HG003 HiFi Revio, GRCh38

downloads.pacbcloud.com/public/revio/2022Q4/HG003-rep1/

PacBio HG004 HiFi Revio, GRCh38

downloads.pacbcloud.com/public/revio/2022Q4/HG004-rep1/
